# Curcumin Effect on Copper Transport in HepG2 Cells

**DOI:** 10.3390/medicina54020014

**Published:** 2018-04-12

**Authors:** Anita Berzina, Inese Martinsone, Simons Svirskis, Modra Murovska, Martins Kalis

**Affiliations:** 1 August Kirchenstein Institute of Microbiology and Virology, Riga Stradins University, Dzirciema 16, Riga LV-1007, Latvia; Anita.Berzina@rsu.lv (A.B.); Simons.Svirskis@rsu.lv (S.S.); Modra.Murovska@rsu.lv (M.M.); 2 Institute of Occupational Safety and Environmental Health, Riga Stradins University, Dzirciema 16, Riga LV-1007, Latvia; inese.martinsone@rsu.lv

**Keywords:** ATP7B, HepG2, curcumin, copper, Wilson’s disease

## Abstract

*Background and Objective*: In Wilson’s disease, copper metabolism is impaired due to defective copper transporting protein ATP7B, resulting in copper accumulation in liver and brain and causing damage to liver and brain tissues. Published data suggest that one of the possible treatments for Wilson’s disease is curcumin—a compound found in the root of *Curcuma longa*. In this study, we tested whether curcumin affects copper transport and excretion in HepG2 hepatocytes carrying wildtype ATP7B. *Materials and Methods*: We examined the impact of 5 µM and 25 µM curcumin on the transport of copper in HepG2 cells incubated with 20 µM and 100 µM CuCl_2_, as well as copper excretion from cells. First, immunofluorescent staining and co-localization analysis were carried out in HepG2 cells using confocal laser scanning microscope and Nikon NIS Elements software. Second, a concentration of copper extracted into cell culture medium was determined using atomic absorption spectrometry. *Results*: The analysis of the co-localization between Golgi complex and ATP7B revealed that both 5 µM and 25 µM doses of curcumin improve the ability of liver cells to transport copper to plasma membrane at 20 µM CuCl_2_, but not at 100 µM CuCl_2_ concentration. However, atomic absorption spectrometry showed that curcumin rather promotes copper absorption into liver cell line HepG2 than excretion of it. *Conclusions*: Curcumin accelerates the transport of copper within liver cells, but does not promote copper excretion from HepG2 cells.

## 1. Introduction

Copper is an important microelement necessary for the functioning of several cellular processes [1,2,3,4]. However, excessive accumulation of copper in tissues leads to oxidative stress, cell death [5,6,7] and damage of cellular proteins, lipids and nucleic acids [8,9]. Copper is absorbed from food by intestinal epithelium ATPase ATP7A [9,10]. Fifteen percent of the absorbed copper is delivered to tissues, while 85% is excreted from the body through gall (98%) or urine (2%), indicating that liver is the main organ for copper homeostasis [11].

Heavy metal transport protein ATP7B [11,12] plays an important role in copper transmembrane transport in hepatocytes. Copper is first bound by CTR1 protein [13,14,15] and then delivered to ATP7B by copper chaperone ATOX1 through copper-dependent protein–protein interaction [13,16] in hepatocytes. Physiological hepatocyte ATP7B transport mechanisms, directly affected by copper, have not been completely explained to date [17].

Mutations in *ATP7B* gene cause Wilson’s disease [11,18], a genetic disease inherited by 1 in 30,000 to 100,000 people [13]. Several biochemical abnormalities are observed in the case of ATP7B defects: less copper is released in liver bile ducts, affecting the binding of copper and ceruloplasmin, and leading to the increase in the free copper levels in blood and subsequent accumulation in tissue causing cell apoptosis [19,20,21]. In Wilson’s disease patients, copper can accumulate in liver, eye cornea, kidneys, brain, etc. [5,6,7]. The accumulation of copper in liver causes different alterations, from small abnormalities in functional liver tests to chronic hepatitis, liver cirrhosis and fulminant hepatitis [9,22].

ATP7B is an essential protein for the maintenance of copper homeostasis and excretion of copper excess from a body. In a cell, ATP7B protein is located in the *trans* part of Golgi complex [9,23]. The protein exercises two functions in liver: (1) it takes part in the copper transport to *trans* Golgi complex where copper binds with ceruloplasmin; and (2) it is involved in copper excretion through gall [9,10]. To perform these functions, membrane ATPases use vesicles for the transport between different cell compartments [24].

Upon the elevation of copper levels in a cell, ATP7B transports it from *trans* Golgi complex to vesicles where copper is stored temporarily. When copper levels exceed 20 µM concentration, the excretion of vesicles containing copper and ATP7B takes place at the apical surface of hepatocytes, and copper is excreted through gall [25].

Curcumin is a hydrophobic polyphenol compound extracted from the roots of *Curcuma longa* plant [26,27]. Curcumin is a common spice and is used as a coloring agent in food and textiles [27,28,29]; however, it is also used in medical applications for the treatment of gall, liver, rheumatic diseases, etc. [27,30,31].

Zhang et al. [32] have found that in Wilson’s disease model of hepatocyte-like cells obtained from fibroblast-derived pluripotent stem cells carrying ATP7B mutation R778L, curcumin was able to partially restore the missing ATP7B copper transport function and correct localization in a cell. However, it was not clear whether curcumin could accelerate copper transport in hepatocytes without Wilson’s disease phenotype.

The aim of this study was to verify whether the experimental settings described by Zhang et al. [32] would also result in a similar mechanism in standard liver hepatoma cell line HepG2. To do that, we tested the effect of different copper and curcumin concentrations (normal and relatively toxic level) on ATP7B localization in non-mutant HepG2 cells as well as the excreted copper levels after curcumin treatment. 

## 2. Materials and Methods

### 2.1. Cell Culturing, Treatment and Sample Collection

HepG2 cells were grown on Poly-l-Lysine-coated (Boster Biological Technology, Pleasanton, CA, USA) glass coverslips in MEM cell culture medium supplemented with 10% FCS, glutamine (2 mM), penicillin and streptomycin (all from Thermo Scientific, Waltham, MA, USA) until they reached 90% confluence. After that, they were pre-treated with 25 µM or 5 µM curcumin (Dr. Elmenstofer GmbH, Ammerbuch-Entringen, Germany) for 24 h and then incubated with 20 µM or 100 µM CuCl_2_ (Alfa Aesan, Karlsruhe, Germany) for 4 h before immunostaining (protocol adapted from Zhang et al. [32]).

For copper measurements, cells were cultured in 25 cm^2^ flasks (BD Falcon, San Jose, CA, USA) until 90% confluence, then treated with 25 µM or 5 µM curcumin for 24 h and then incubated with 100 µM or 20 µM CuCl_2_ for additional 24 h, respectively. After that, cells were washed with PBS and fresh MEM medium added. Samples for atomic absorption spectrometry measurements were collected after 1.5, 3, 4.5, 6, 7.5 and 9 h and cells counted using Bürker chamber and standard Trypan Blue (Sigma Aldrich, Steinheim, Germany) staining for evaluation of dead cell proportion at the end of the experiment. Before temporary freezing, medium samples were centrifuged to remove cell debris.

### 2.2. Immunofluorescent Stainings, Confocal Microscopy and Co-Localization Analysis

Cells were fixed with 4% formaldehyde (Sigma Aldrich, Steinheim, Germany), immunostained with primary antibodies (Golgi complex marker: mouse α p230 (Becton Dickinson, Franklin Lakes, NJ, USA) at 1:1000 dilution and rabbit α ATP7B (Proteintech, Chicago, IL, USA) at 1:500 dilution), and incubated overnight at +4 °C. Secondary polyclonal antibodies included Goat α Mouse IgG (H + L), Alexa Fluor 488 (AbCam, Cambridge, MA, USA) and Donkey α Rabbit IgG (H + L), Dylight 550 (AbCam, Cambridge, MA, USA), diluted at 1:500, and were incubated for 1 h at room temperature. Fluoroshield mounting medium with DAPI (Sigma Aldrich, Steinheim, Germany) was used.

The samples were analyzed with Nikon A1R+ (Nikon, Tokyo, Japan) confocal microscope using lasers with 405 nm, 488 nm and 561 nm wavelengths. A 100× oil immersion objective with numerical aperture 1.45 (Nikon, Japan) was used.

NIS Elements (Nikon, Japan) software was used for co-localization analysis. The co-localization analysis between Golgi complex marker p230 ant ATP7B was carried on following method described by Manders [33]. Results were expressed using Manders overlap coefficient. Number of pictures *n* = 3 was considered the minimal number of replication for data analysis.

### 2.3. Atomic Absorption Spectrometry Measurements

The analysis was performed using atomic absorption spectrometer Varian Spectra AA 200 Z (Varian, Palo Alto, CA, USA) with graphite furnace and electrothermal atomizers as better described in [34,35]. Zeeman background correction at copper-specific wavelength (Cu—327.4 nm) was applied. Values were normalized to the number of cells in each sample. Medium taken from cells not exposed to copper was used as a negative control accounting for the basal level of copper released by HepG2 cells.

### 2.4. Statistical Analysis

All the graphs, calculations, and statistical analyses were performed using GraphPad Prism software version 7.0 for Mac (GraphPad Software, San Diego, CA, USA). To test whether the collected numerical data are normally distributed, the D’Agostino and Pearson and Shapiro-Wilk normality tests were applied. The comparison of means between different groups of numerical variables was performed using one-way or two-way ANOVA. Homogeneity of variances was tested using Brown-Forsythe and Bartlett’s tests. If data were not normally distributed, the comparison of medians between different groups was switched to non-parametric one-way ANOVA on ranks or Kruskal-Wallis test followed by post-hoc analysis. Changes of measured copper and differences among the groups were assessed with RM two-way ANOVA (considering two main sources of variation—time and treatment) followed by two-stage step-up method of Benjamini, Krieger and Yekutieli as post-hoc test. Influence of curcumin was assessed by Multiple t tests with correction for multiple comparisons using Holm-Sidak method. Results are expressed as median and interquartile range (IQR) as dispersion characteristic. *p*-Value less than 0.05 (*p* < 0.05) was considered as statistically significant.

## 3. Results

**Figure 1:**
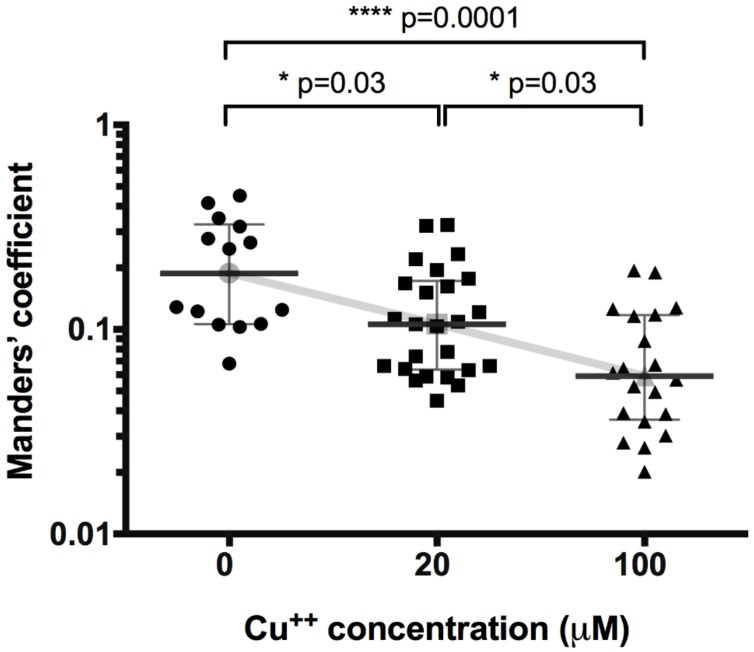
Effect of copper concentration on co-localization of Golgi complex protein p230 and ATP7B. Data are displayed as scatter graph with median and interquartile range (IQR); Kruskal–Wallis test (K-W statistic 16.4, *p* = 0.0002), between group comparison by two-stage step-up method of Benjamini, Krieger and Yekutieli as post-hoc test. Gray line connects medians.

We performed a co-localization study between ATP7B and p230 to test the translocation of ATP7B away from Golgi complex in HepG2 cells upon copper treatment. First, we tested the basic effect of increasing copper concentrations on translocation of ATP7B (Figure 1). The co-localization of ATP7B with Golgi complex marker p230 decreased from the value of 0.19 (0.11–0.32) (number of images *n* = 14) detected in not exposed cells to 0.11 (0.06–0.17) (*n* = 25) and 0.06 (0.04–0.12) (*n* = 20) at 20 μM and 100 μM CuCl_2_, respectively (at 20 μM *p* = 0.03, at 100 μM *p* < 0.0001).

**Figure 2:**
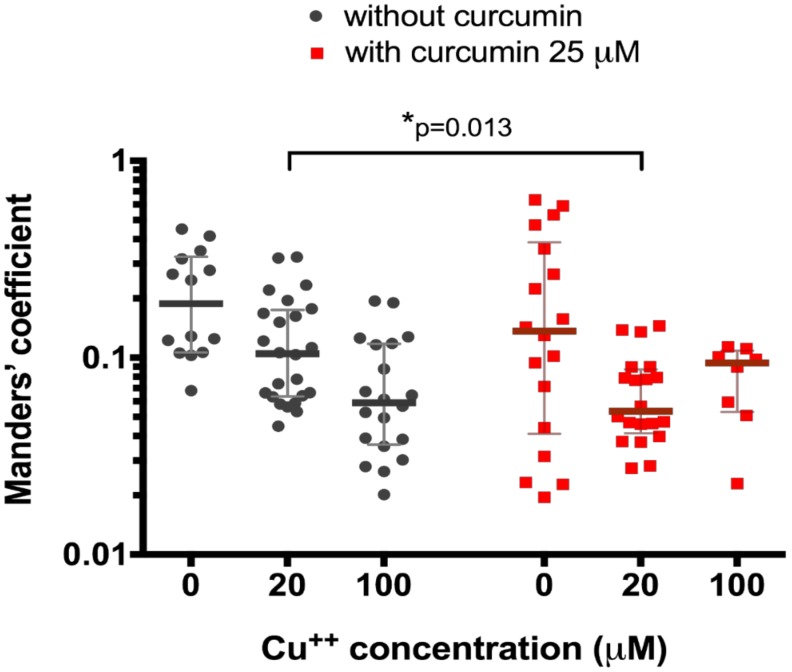
Co-localization of ATP7B and Golgi complex marker p230 with or without pre-treatment with 25 µM curcumin in HepG2 cells incubated in the culture medium with different copper concentration. Data are displayed as scatter graph with median and interquartile range (IQR); *p*-value adjusted with approach of Multiple t-tests for grouped data with correction for multiple comparisons using Holm-Sidak method.

We then tested the effect of 25 μM curcumin treatment on the 20 μM and 100 μM CuCl_2_-treated HepG2 cells. We registered significantly different (0.05 (0.04–0.09) co-localization, *n* = 20, vs. 0.11 (0.06–0.17), *n* = 25, *p* = 0.013, Figure 2) in curcumin-preincubated cells only at CuCl_2_ concentration of 20 μM (0.09 (0.05–0.11), *n* = 8, vs. 0.06 (0.04–0.12) (*n* = 20), *p* = 0.97, Figure 2).

**Figure 3:**
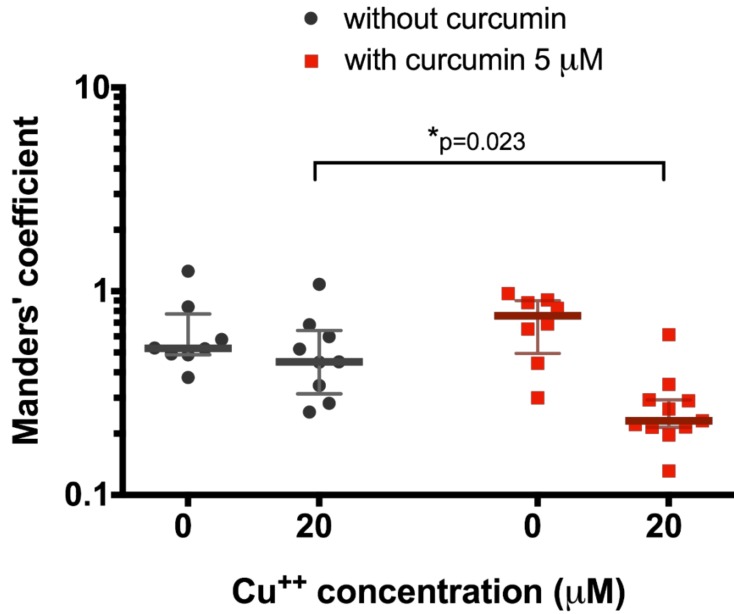
Co-localization of ATP7B and Golgi complex marker p230 with or without pre-treatment with 5 µM curcumin in HepG2 cells incubated in the culture medium with different copper concentration. Data are displayed as scatter graph with median and interquartile range (IQR); *p*-value adjusted with approach of Multiple t-tests for grouped data with correction for multiple comparisons using Holm–Sidak method.

After that, we decided to test the effect of a lower curcumin concentration (5 μM) on 20 μM copper-treated HepG2 cells since it had been shown as a relevant concentration at which curcumin could start to affect ATP7B translocation. In this case, we also observed significant decrease in ATP7B co-locallization with Golgi complex in cells exposed to 20 μM CuCl_2_ when pre-incubated to 5 μM curcumin (0.23 (0.21–0.29), *n* = 10, vs. 0.45 (0.31–0.64), *n* = 9, *p* = 0.0187, Figure 3).

**Figure 4:**
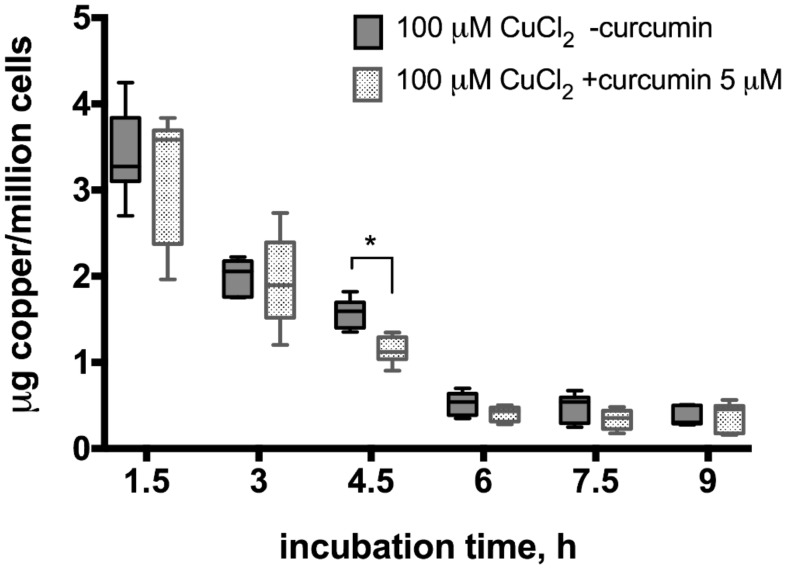
Copper levels in culture medium after incubation of HepG2 cells with curcumin (0 or 5 µM) and CuCl_2_ (100 µM). The atomic absorption spectrometry measurements were adjusted for background values of the cell culture medium, making 0 h reference measurement to equal 0 µg copper/million cells after measuring copper-free HepG2 medium in control experiments (data not shown). Curcumin concentration 5 µM. Data are displayed as box-plot graph with median, interquartile range (IQR) and range min-max; two-way RM ANOVA (time, F (5.80) = 293.1 (*p* < 0.0001); treatment, F (1.16) = 3.49 (*p* = 0.08), between group comparison by two-stage step-up method of Benjamini, Krieger and Yekutieli as post-hoc test; * *p* < 0.05.

**Figure 5:**
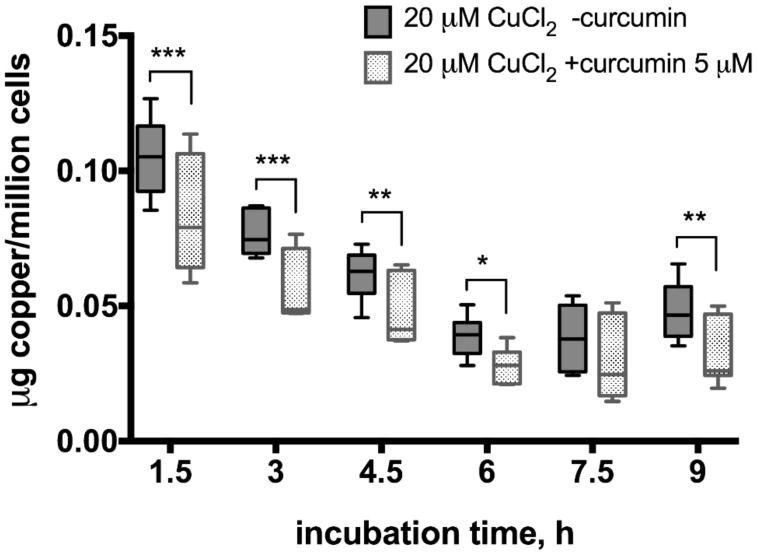
Copper levels in culture medium after incubation of HepG2 cells with curcumin (0 or 5 µM) and CuCl_2_ (20 µM). The atomic absorption spectrometry measurements were adjusted for background values of the cell culture medium, making 0 h reference measurement to equal 0 µg copper/million cells after measuring copper-free HepG2 medium in control experiments (data not shown). Curcumin concentration 5 µM. Data are displayed as box-plot graph with median, interquartile range (IQR) and range min-max; two-way RM ANOVA (time, F (5.80) = 246.2 (*p* < 0.0001); treatment, F (1.16) = 9.15 (*p* = 0.0081), between group comparison by two-stage step-up method of Benjamini, Krieger and Yekutieli as post-hoc test; * *p* < 0.05, ** *p* < 0.01, *** *p* < 0.001.

To evaluate the potential curcumin effect on copper excretion from hepatocytes, we performed atomic absorption spectrometry measurements in samples collected after incubation of cells exposed to 100 or 20 µM CuCl_2_ with or without 5 µM curcumin pre-incubation (Figure 4 and Figure 5). When cells were incubated with 100 µM CuCl_2_, we observed rapid excretion of copper as measured 1.5 h after cell washing and medium change, after which the concentration of copper gradually decreased in the medium (Figure 4). At all time points, except 4.5 h, there were no significant differences between curcumin-treated and control samples (Figure 4).

In the 20 µM CuCl_2_-treated samples, a smaller increase in copper levels was observed right after the medium change which dropped until 6 h but then started to increase again (Figure 5). Moreover, the excreted copper levels differed significantly between curcumin-treated and non-treated samples, except 7.5 h time point when the gradual decrease of copper levels in the medium stopped and started to increase again (Figure 5).

## 4. Discussion

Our observation of ATP7B translocation away from Golgi complex to cytoplasmic vesicular compartment upon elevated copper levels (Figure 1) is consistent with data in literature [3,36,37,38,39]. This indicates that ATP7B function is similar in different cell lines and organisms. However, almost nothing is known about the curcumin effect on ATP7B function: such data in literature are missing. In 2009, van den Berghe et al. found that curcumin can partially restore function of several human ATP7B mutants in HEK293T cells [40]. Later, Zhang et al. demonstrated that curcumin can improve ATP7B function carrying Wilson’s disease mutation in murine induced pluripotent stem cell-derived hepatocyte-like cells [32]. However, since these are the only publications, it has not been clarified whether curcumin has an effect on wild-type ATP7B function in healthy human hepatocytes. Therefore, we chose HepG2 cell line as a model for human hepatocytes with intact copper transport and we treated the cells with different copper concentrations and different curcumin doses.

At first, we used relatively high curcumin dose, 25 μM (Figure 2). The results show that curcumin enhances ATP7B translocation only at medium non-toxic [41] copper concentration, 20 μM. At a relatively toxic [41] 100 μM copper level, there is a decrease in co-localization of ATP7B and p230, which is induced by copper alone and not enhanced by curcumin. Thus, we conclude that curcumin cannot affect copper transport in cells treated with toxic copper levels but can contribute to ATP7B-mediated copper transport to plasma membrane at relatively medium copper levels.

However, there are published data that curcumin concentration over 20 μM can cause cytotoxic effect [42,43,44], thus it should not be used clinically as a treatment agent at this dose. Therefore, although we did not observe curcumin toxicity in our experiments with HepG2 cells, we also tested curcumin effect on 20 μM CuCl_2_-treated cells at a concentration of 5 μM. We demonstrated that, even at 5 μM dose, curcumin can improve ATP7B-mediated copper transport in cells making it suitable for testing in vivo, followed by clinical trials in humans, since the dose is not cytotoxic.

To estimate the curcumin effect on copper excretion from liver cells, we performed repeated, time-controlled atomic absorption spectrometry measurements of copper in cell culture medium. We observed that HepG2 cells treated with both 100 and 20 μM CuCl_2_ released copper into cell culture medium, which might be explained by medium change (Figure 4 and Figure 5). In the 100 μM CuCul_2_, copper re-absorption rate was similar in curcumin-treated and non-treated cells confirming that curcumin has no effect on cells treated with toxic copper levels (Figure 4). In contrast, curcumin-treated cells showed significantly faster copper re-absorption at lower copper levels (Figure 5) as indicated by significantly lower medium copper levels at each time-point. After re-absorption of copper into the 20 μM CuCl_2_-treated cells had stopped at 7.5 h, cells started to excrete copper again (Figure 5). Significantly less copper was excreted in the curcumin-treated cells. Thus, although curcumin stimulates intracellular copper transport, it results in more copper accumulated in HepG2 cells.

Despite the lack of research on curcumin potential to influence copper transport in hepatocytes, a plethora of data has been collected on different biological properties of curcumin. A variety of curcumin properties has been listed in relation to different diseases [45,46,47,48,49]. Naturally, we may inquire whether the effects of curcumin on copper transport are related to some of the mechanisms described. Obviously, the antioxidant potential of curcumin may not be involved since this state is present only during copper deficiency, not toxicity [50]. The reduction of lipid accumulation and cross-talk with immune system could be considered in vivo experiments but not in our in vitro hepatoma cell line model where intracellular effect of curcumin on copper transport and export was demonstrated. However, indirect curcumin effect through secondary messenger pathways cannot be excluded, as pro-inflammatory cytokine expression can be altered through NF-κB [51]. Additional studies might clarify exact mechanisms involved in the positive effect of curcumin on ATP7B function—it is the chaperone effect, indirect NF-κB action or some other mechanism which results in improved copper transport.

We suggest that, although curcumin has shown positive effect on stimulation of copper excretion from induced pluripotent and embryonic stem cell-derived liver-like cells [32], there is a different copper absorption/excretion balance in hepatocyte cell-line HepG2. We observed more copper being absorbed than excreted at the given copper levels in HepG2 cells. Curcumin is contributing to a faster absorption rate of copper under these conditions. In our experimental conditions, we observe that hepatocytes are more accumulating copper than excreting it. The initial copper excretion peak observed at 1.5 h measurement is most likely the consequence of a shock after changing medium for copper-free medium and dramatic drop of copper concentration gradient on plasma membrane. However, the precise mechanism responsible for such an effect of curcumin on copper transport must be studied further.

## 5. Conclusions

Curcumin at both 25 and 5 μM doses accelerates intracellular copper transport from Golgi complex to cytoplasm in HepG2 hepatocytes. This effect is observed at 20 μM, but not at the relatively toxic 100 μM copper level. In HepG2 cells, rather copper absorption than excretion takes place at 20 μM and 100 μM copper concentrations. Moreover, curcumin accelerates absorption of copper into HepG2 cells unless the cells have already absorbed toxic levels of copper.
